# Novel Strategies on Personalized Medicine for Breast Cancer Treatment: An Update

**DOI:** 10.3390/ijms18112423

**Published:** 2017-11-15

**Authors:** Carmen W. H. Chan, Bernard M. H. Law, Winnie K. W. So, Ka Ming Chow, Mary M. Y. Waye

**Affiliations:** The Nethersole School of Nursing, The Chinese University of Hong Kong, Shatin, The New Territories, Hong Kong, China; whchan@cuhk.edu.hk (C.W.H.C.); bernardlaw@cuhk.edu.hk (B.M.H.L.); winnieso@cuhk.edu.hk (W.K.W.S.); kmchow@cuhk.edu.hk (K.M.C.)

**Keywords:** breast cancer, personalized medicine, cancer treatment, quality of life

## Abstract

Breast cancer is the most common cancer type among women worldwide. With breast cancer patients and survivors being reported to experience a repertoire of symptoms that are detrimental to their quality of life, the development of breast cancer treatment strategies that are effective with minimal side effects is therefore required. Personalized medicine, the treatment process that is tailored to the individual needs of each patient, is recently gaining increasing attention for its prospect in the development of effective cancer treatment regimens. Indeed, recent studies have identified a number of genes and molecules that may be used as biomarkers for predicting drug response and severity of common cancer-associated symptoms. These would provide useful clues not only for the determination of the optimal drug choice/dosage to be used in personalized treatment, but also for the identification of gene or molecular targets for the development of novel symptom management strategies, which ultimately would lead to the development of more personalized therapies for effective cancer treatment. In this article, recent studies that would provide potential new options for personalized therapies for breast cancer patients and survivors are reviewed. We suggest novel strategies, including the optimization of drug choice/dosage and the identification of genetic changes that are associated with cancer symptom occurrence and severity, which may help in enhancing the effectiveness and acceptability of the currently available cancer therapies.

## 1. Introduction

Breast cancer is currently the most prevalent cancer type among women worldwide. In 2012, more than 1.6 million new cases of breast cancer were reported, and it had resulted in more than 500,000 deaths [1]. Collectively, breast cancer can be classified into several sub-types based on the observed presence of certain breast cancer-associated biomarkers, such as estrogen receptor (ER), progesterone receptor (PR), Ki-67 (a protein marker with prognostic and predictive potential for adjuvant chemotherapy), and human epidermal receptor 2 (HER2), in the tumors (Table 1) [2]. In light of the high and increasing prevalence of breast cancer, the development of effective treatment strategies for breast cancer is warranted. Currently, the strategies that are used to treat breast cancer include chemotherapy, radiation therapy, hormonal therapy, and surgery, although immunotherapy, the use of monoclonal antibodies (mAbs), which is known to interfere with certain cancer-related molecular/signaling pathways in cancer treatment, is increasingly utilized. Furthermore, with the development and increased use of targeted therapies, which involve the use of therapeutic molecules that would specifically modulate the pathways implicated in tumor progression, the survival rate of breast cancer patients and their patient outcomes have improved over the past years [3]. However, oncologists still face challenges in the implementation of efficacious breast cancer treatment through targeted therapies, primarily due to the progressive development of therapeutic resistance by the tumors, which consequently leads to patient relapses [4]. Besides, certain cancer deaths may in fact be attributed to the detrimental effects of cancer treatment. A study in the United Kingdom revealed that about 2.5% of breast cancer patients who underwent systematic anticancer therapy died within 30 days of receiving the therapy [5]. Likewise, a population-based study also revealed that the 30-day mortality rate of a cohort of patients of various cancer types as a result of receiving palliative radiotherapy even reached 12.3% [6]. In addition, the immense cost that is required to receive such therapies, owing to the use of expensive therapeutics, has also imposed considerable economic burden to the patients’ families. In light of this, the development of further strategies in developing effective cancer treatment is of crucial importance. Over the past decade, personalized medicine has gained increasing attention in terms of its prospect in more effective cancer treatment and management.

With the advances in the development of high-throughput DNA sequencing and bioinformatics analyses, the prospect of the use of personalized medicine has become increasingly realistic. As a result of the increased utilization of personalized medicine in recent years, the overall survival of cancer patients has generally been improved. This improvement was shown to be more prominent among metastatic breast cancer patients when compared to patients with colorectal cancer and metastatic non-small cell lung cancer [7]. Nevertheless, not all patients are able to benefit from personalized treatment and oncologists are still facing many challenges in its implementation. To date, some barriers to the successful implementation of personalized treatment among breast cancer patients have been suggested. These include cancer heterogeneity [8,9,10], and variations in the level of responses to different cancer treatment regimens (chemotherapy, radiation, or surgical treatments) among individuals that are currently not fully understood [11,12]. Nevertheless, it was cited that the heterogeneity of the single nucleotide polymorphisms (SNPs) in certain cancer-associated genes (such as genes coding for cytochrome P450 variants) that are possessed by different ethnic groups could be a potential factor for the variations in treatment response among individuals [13]. Such variations would impose challenges on the development of personalized therapies, where the choice of therapeutic drugs/molecules that are used for therapies and their dosages have to be specifically tailored to an individual. Specifically, the formulation of the optimal personalized therapy for different individuals possessing various SNPs is going to be costly and tedious due to the need to tailor the therapies for multiple genes with some still unidentified SNPs to date, based on the modified treatment responses of various individuals caused by these SNPs. Further research into personalized medicine for breast cancer patients should therefore address these challenges.

Previously, a number of reviews have been published providing an overview on the variety of therapies available during the initial treatment of breast cancer [14,15,16,17]. However, reviews that provide suggested strategies on how to improve on the current therapies, in terms of enhancing effectiveness and the reduction of side effects, are currently lacking. This review attempts to highlight some of the recent research that would provide clues to the exploration for new options in the development of personalized therapies available for breast cancer patients and survivors, which would potentially enhance the effectiveness and acceptability of the currently available cancer therapies.

## 2. Side Effects of Current Cancer Therapies Reduce the Quality of Life (QOL) of Breast Cancer Patients and Survivors

Current cancer therapies, including chemotherapy, are known to induce undesirable cancer-associated symptoms among breast cancer patients, which are detrimental to the QOL of these patients [18,19]. A previous study had evaluated the symptom experience of breast cancer patients who are undergoing chemotherapy, and they identified 38 common symptoms among these individuals, in which they were classified into five stable symptom clusters (psychological, hormonal, nutritional, gastrointestinal, and epithelial) [20]. In another study, the authors investigated the effect of chemotherapy on symptom experience and QOL of breast cancer survivors who received chemotherapy [21]. It involved an assessment among these individuals for their mental and physical QOL at various time points, including before chemotherapy, after cycle 3 of chemotherapy, within 2–3 weeks of completing adjuvant chemotherapy, and at least eight years after chemotherapy. Though no obvious decline in cognitive function was observed, the authors reported that patients experienced an increase in the severity of depressive symptoms and fatigue. Therefore, breast cancer patients undergoing chemotherapy normally experience multiple symptoms, which was suggested to exhibit a synergistic effect on patient outcomes [22,23]. Another point of note is that cancer patients at different cancer stages may possess different perceptions on the need in addressing these detrimental treatment-associated side effects. For example, these side effects may appear more acceptable to early-stage cancer patients, due to the perceived brighter prospect of the treatment in curing the disease at an early stage. In contrast, such side effects on patients with metastatic tumors should be more aggressively addressed as metastatic cancers are generally incurable and that ensuring a better QOL of these patients is of greater importance. Therefore, a better understanding on the common treatment-related side effects among advanced breast cancer patients and survivors is required, so as to provide clues to the development of strategies in tailoring treatment regimens that would effectively alleviate these side effects.

Overall, previous studies had identified a repertoire of more devastating symptoms that are experienced by these patients that would exhibit a more far-reaching impact, as described below.

### 2.1. Premature Menopause or Chemotherapy-Induced Menopause (CIM)

Breast cancer patients undergoing chemotherapy are known to be at an increased risk of premature menopause, resulting in menopausal symptoms that would decrease their QOL [24]. The incidence rate of premature menopause after chemotherapy of young breast cancer patients was reported to be 13.3% [25]. Although premature menopause might have been perceived to be less harmful among women with children, such a condition could predispose them to an increased risk of cardiovascular diseases [26]. Indeed, female cancer survivors who underwent cardio-toxic therapy and have experienced premature menopause appear to be at an increased risk of cardiac morbidity [27]. Premature menopause may also contribute to bone fragility, and could lead to spontaneous rib fractures [28]. Thus, interventions through exercise could be a useful strategy for breast cancer patients undergoing chemotherapy to reduce cardiovascular disease risk [29]. Besides the increase in long-term mortality risks as a result of premature menopause, women often experience unpleasant symptoms that reduce their QOL. Thus, even though ovary ablation (OA) was shown to reduce breast cancer relapse (in combination with the use of either tamoxifen or aromatase inhibitors) in high-risk women, adjuvant OA should still be utilized with careful considerations [30]. Overall, CIM is one of the treatment-associated symptoms for breast cancer patients that would lead to increased risk of further complications. Interventions that reduce the risk of these complications should be made available to these patients who are undergoing chemotherapy.

### 2.2. Chemotherapy-Induced Peripheral Neuropathy (CIPN)

Chemotherapy-induced peripheral neuropathy (CIPN) may occur in many breast cancer patients during treatment, or in some cases many years after they complete their treatment. One study reported that up to 45% of breast cancer survivors would still feel numbness in their peripheral limbs, a symptom that is associated with CIPN, six years after chemotherapy [31]. Patients with CIPN are more susceptible to falls, and therefore they are at increased risk of bone fractures [32]. Nevertheless, a Danish study found that peripheral neuropathy did not affect the relative dose intensity in the treatment of their patients with adjuvant chemotherapy [33], indicating that the experience of CIPN among breast cancer patients would not affect the course of cancer treatment, despite its detrimental effect on their locomotion.

### 2.3. Cognitive Dysfunction

Cognitive dysfunction is one of the treatment-associated symptoms that affects mainly older cancer patients [34]. One study [35] demonstrated that up to 46% of patients aged 65 or above, who were admitted for breast, prostate, or colorectal cancer, suffered from cognitive impairment. The authors also showed that patients who were determined to exhibit cognitive impairment using the Montreal Cognitive Assessment (MoCA > 26) were found to be at a higher risk of death than those who did not [35]. Although the exact mechanism of how chemotherapy can induce cognitive dysfunction is not known, previous studies suggested that neuroinflammation and oxidative stress might play a major role [36].

### 2.4. Depression

Depressive symptoms were previously reported in breast cancer patients who underwent chemotherapy. For example, mild to moderate levels of depression was observed in over one half of breast cancer survivors in one study, and their depressive symptoms were found to be accompanied by cognitive dysfunction [21]. Further, in an Indian study, 22% of breast cancer survivors were found to exhibit moderately severe to severe levels of depression. This condition was also demonstrated to be associated with a poorer QOL among these patients [37,38].

### 2.5. Pain

Pain is one of the most well-established symptoms that is suffered by cancer patients undergoing chemotherapy, primarily due to the occurrence of CIPN, where alterations in neurotransmissions and actions of pro-inflammatory cytokines in peripheral nerves are believed to be the major causes [39]. Moreover, a recent meta-analysis revealed a number of risk factors that may increase the odds of experience of pain among breast cancer survivors [40]. These include obesity, lower level of education, lymphedema, non-smokers, axillary lymph node dissection, and undertaking chemotherapy, hormonal therapy, and radiation therapy. The finding that undertaking cancer therapies is a risk factor of pain provides further evidence for the direct effect of cancer treatment on the experience of pain among breast cancer patients.

## 3. Recommended Strategies of Personalized Medicine Development in Improving the Efficacy of Chemotherapy

Owing to the unpleasant symptoms associated with chemotherapy described above, strategies that are effective in improving the efficacy of cancer treatment need to be developed in order to shorten the required duration of the treatment process. Nevertheless, as discussed, variations in the level of response to cancer treatment between patients do exist. Indeed, both patients’ age and cancer stage were suggested to affect their response to cancer treatment. For example, older patients tend to be more vulnerable to the detrimental effects that are exerted by chemotherapeutic drugs due to alterations in pharmacokinetics with advancing age, resulting in poorer drug clearance [41]. Likewise, low complete response rate to chemotherapy was also observed among patients with more advanced cancer [42]. Therefore, the optimal drug choice/dosage to be used in cancer treatment needs to be determined for each individual patient in order to ensure that the treatment would be safe and effective for every patient, and this remains to be one major challenge for oncologists and physicians. Here, we describe the possible strategies that can be implemented in order to optimize the drug choice/dosage used in cancer therapies and enhance their effectiveness.

### 3.1. Use of Gene Expression Profiling Techniques in the Optimization of Drug Choice for Cancer Treatment

In this section, we provide an overview of previous studies demonstrating the strategies of personalized therapies that are available to improve treatment responses, based on our knowledge on the genes that are linked to cancer development and altered responses to cancer therapies.

De Bruin et al. [43] has recently demonstrated an effective methodology in detecting the glutamic acid-to-lysine mutation at position 17 in *AKT1* (*AKT1* E17K mutation), a mutation that is of low prevalence among cancer patients, in tissue and plasma samples of advanced cancer patients. With AKT being a critical component of the cancer-promoting phosphoinositide 3 kinase-AKT-mammalian target of rapamycin (PI3K-AKT-mTOR) pathway [44], development of inhibitors targeting this pathway could serve as a promising strategy for the inhibition of tumor progression. This was demonstrated by the effectiveness of the use of chemotherapeutic drugs that target the PI3K-AKT-mTOR pathway, such as everolimus in breast cancer treatment [45]. Although *AKT1* E17K mutation is rare, it was demonstrated to be a driver mutation for breast cancer [46], thereby suggesting the potential of the E17K mutation as a diagnostic mutation for breast cancer. Therefore, the development of a reliable method for the detection of this rare mutation is warranted for the assessment of clinical activity of cancer therapies that can be personalized for breast cancer patients. De Bruin et al. had developed and validated a companion diagnostic assay for the detection of *AKT1* E17K mutation using a competitive allele-specific TaqMan polymerase chain reaction (PCR) assay in formalin-fixed paraffin-embedded tumor tissues or plasma specimens in cases of circulating tumor DNA. This newly developed technique would therefore enable the more reliable and accurate testing of the clinical activity and responses of certain breast cancer therapies in patients, including the “endocrine (fulvestrant) therapy”, combined with the use of AZD5363, the pan-AKT inhibitor. This would provide useful clues to the optimization and tailoring of such therapy specifically for breast cancer patients.

Andersen et al. [47] had previously reported the feasibility for using phospho profiling to identify biomarkers that are targeted by PI3K-AKT-mTOR pathway inhibitors. The study identified a number of phosphoproteins, which their abundance would be altered in response to these inhibitors. The identification of these phosphoproteins as biomarkers of the PI3K-AKT-mTOR pathway inhibitors would therefore enable the determination of the efficacy of these inhibitors in PI3K pathway (and therefore breast cancer) inhibition, based on the level of phosphorylation of these biomarkers. This in turn would provide a platform for the development of personalized therapy for breast cancer patients through the determination of the optimal choice of the PI3K-AKT-mTOR pathway inhibitors to be used in cancer treatment. Such optimization is of crucial importance especially due to the possibility of the development of drug resistance that is caused by the activation of compensatory pathways in response to the drugs used in cancer therapies, which drastically reduce their efficacy. Indeed, it was previously reported that although the use of PI3K-AKT-mTOR pathway inhibitors and inhibitors of other molecular pathways implicated in breast cancer (such as cyclin-dependent kinase 4/6 signaling pathway) had demonstrated being effective in prolonging progression-free survival of breast cancer patients, an increase in overall survival was generally not observed [48]. The inability of these therapies in prolonging overall survival therefore prompts the development of a more personalized approach in the prescription of cancer treatment through the optimization of drug choice.

Recently, Turnbull et al. had developed a model in the identification of the biomarkers specific to letrozole, an aromatase inhibitor, among breast cancer patients [49], thereby contributing to the further identification of drug-specific biomarkers for the development of personalized cancer therapies. The authors identified two genes that were upregulated in response to letrozole treatment—one implicated in immune responses (*IL6ST*) and the other implicated in apoptosis induction (*NGFRAP1*). In contrast, two genes that were implicated in cellular proliferation (*ASPM* and *MCM4*) were also identified to be downregulated post-treatment. These identifications provide further options of biomarkers that can be utilized for the optimization of drug choice in the development of personalized cancer treatment.

Moreover, detection of the overexpression of genes implicated in breast cancer had also been suggested to be useful in the development of personalized treatment for breast cancer patients. For example, overexpression of human epidermal growth factor receptor 2 (HER2) had long been considered to be associated with breast cancer progression, and the use of chemotherapeutic drugs that target this protein, such as trastuzumab, had shown promise in treating patients bearing HER2-overexpressing tumors [50]. Nevertheless, a recent study demonstrates that the overexpression status of HER2 could change over various stages across the cancer trajectory [51], and therefore planning of trastuzumab therapy cannot be based entirely on the receptor status in primary tumors. The authors demonstrated that the rate of expression of HER2 can be increased by about 20% when the breast tumor progressed from a primary tumor state to the metastasized state [51]. In light of this, prior to the planning for trastuzumab therapies, an assessment of HER2 overexpression status in tumors should be performed. For example, repeat biopsy could be an option for assessing HER2 status prior to the commencement of therapies [52], in order to minimize the potential effect of any discordances in HER2 status between primary and metastatic breast tumors on treatment effectiveness. Furthermore, the development of various molecular imaging technologies for the detection of HER2 in tumors would provide a useful means for such assessment [53]. Such a personalized approach in the administration of trastuzumab therapies would likely increase the efficacy of the treatment process.

Another useful genomic biomarker that may be used to evaluate the clinical efficacy of cancer therapies is mitochondrial DNA (mtDNA) common deletions. mtDNA common deletions were known to be a type of mtDNA mutation that occur frequently in breast cancer patients [54]. Such mutations are believed to be the result of oxidative damages, which are caused by the high level of reactive oxygen species (ROS) that are generated during oxidative phosphorylation in the mitochondria [55]. Indeed, previous studies had indicated the role of mtDNA alterations in chemotherapeutic drug resistance and therefore outcomes of cancer therapies [56,57]. Therefore, the utilization of mtDNA mutations/deletions could serve as a biomarker for the optimization of the current cancer therapies.

Interestingly, in addition to genomic alterations, alterations in the metabolome were also suggested to be a useful biomarker for predicting cancer occurrence, and hence for the development of personalized therapy. Hosokawa et al. recently demonstrated that the level of phosphatidylcholine (32:1) in triple-negative breast cancer patients with disease recurrence was significantly higher than those without [58]. In another study, increased seral levels of testosterone was shown to be associated with increased risk of breast cancer recurrence [59]. The findings demonstrating the role of these metabolites in affecting breast cancer recurrence would certainly spark interest on further research in whether hormonal therapies against cancer can be tailored using a personalized approach. For example, based on the finding that testosterone levels are associated with breast cancer recurrence, Secreto et al. suggested the regular measurement of testosterone levels during the course of hormonal therapy using gonadotropins-releasing hormone analogues, which are molecules that reduce testosterone production. The therapy would then be administered whenever the seral level of testosterone level has risen. This personalized approach in hormonal therapy administration would therefore ensure that the seral testosterone levels of patients can be kept in check, thereby reducing the risk of cancer recurrence.

### 3.2. Monitoring of Circulating Tumor Cells

While genetic biomarkers can be identified through gene expression profiling, molecular biomarkers, which are primarily present in circulating tumor cells can be identified through immunocytochemistry using fluorescent antibody staining. For example, Pachmann et al. described a technique involving the immunocytochemical staining to monitor the changes in the number of circulating epithelial tumor cells (CETC) in breast cancer patients [60]. They showed that patients exhibiting an increase in numbers of CETC would be at an increased risk (fivefold or more) of relapse when compared to those showing a decrease or displaying no change in CETC numbers, indicating that CETC is implicated in breast cancer recurrence and that the number of CETC could serve as a biomarker for monitoring disease recurrence among breast cancer survivors.

Further, genomic changes occurring in circulating tumor cells may also provide clues to the prediction of treatment outcomes. Indeed, whole genome sequencing (WGS) of circulating tumor cells was suggested to be a useful tool in providing information on the optimal type of pharmacological interventions in treating cancer, due to the effectiveness of this technique in identifying the “driver mutations” of the cancer and therefore the genes that are involved in tumor progression [61]. For example, Wheler et al. revealed that the simultaneous occurrence of the amplification of genes involved in the fibroblast growth factor (FGF)/FGF receptor and those within the PI3K/AKT/mTOR pathway would result in higher response rates to therapies utilizing chemotherapeutic drugs [62]. Overall, the status of the circulating tumor cells would provide useful information on guiding the development of personalized therapies, based on their numbers that are present in serum and the mutations they possess.

### 3.3. Use of Pharmacogenomics in Predicting Response to Chemotherapeutic Drugs

Pharmacogenomics, a study on how differences in genes can contribute to modifications in drug response, would also provide useful information on how pharmacological interventions should be tailored in personalized approaches. Indeed, certain genetic polymorphisms were shown to affect the metabolism and transport of chemotherapeutic drugs. For example, tamoxifen, a chemotherapeutic drug for breast cancer treatment, is required to be metabolised into endoxifen via a variant of the cytochrome P450 enzyme called cytochrome P450 2D6 (CYP2D6) before the drug can exhibit its effects [63]. Therefore, individuals with various polymorphism in the cytochrome *P450* gene will exhibit considerable variability in the experience of the severity of side effects that is caused by tamoxifen intake. Consistent with this, variants of the *CYP2D6* gene were associated with the discontinuation of tamoxifen therapy, due to the experience of side effects [64]. Further, another cytochrome P450 enzyme, CYP2C9, was shown to contribute to variations in steady-state endoxifen levels, albeit in a minor manner [65]. All of these findings suggest that genetic variations should be taken into account during the planning of personalized therapies for breast cancer patients in order to ensure that an optimal drug dosage is used during cancer treatment. They also highlight the value of the potential application of knowledge in pharmacogenomics to the successful implementation of personalized medicine. Nevertheless, the use of pharmacogenomics in guiding the development of personalized therapy is still in need of further validation, as evidence for the clinical utility of tamoxifen is yet to be confirmed by large-scale clinical studies examining the beneficial effect of tamoxifen on patients with various CYP2D polymorphisms.

### 3.4. Use of MicroRNA in Triggering Drug Release

MicroRNA are non-coding RNA that are present in the genome, and it plays a major role in the control of gene expression through RNA silencing [66]. Recently, microRNA has been shown to trigger the release of chemotherapeutic drugs that are delivered by nanoparticles to cells [67]. The authors treated breast cancer cell lines with nanocarriers that were complexed with doxorubicin, and upon the presence of microRNA21 in cells, the drugs were able to be released from the nanocarriers and exhibit their chemotherapeutic effects, such as the decrease in cancer cell viability. This finding therefore provides the basis for the development of a new technique where chemotherapeutic drugs can exhibit their effect on-demand, which would be useful in limiting the side effects that are caused by the drugs. Further development of such a technique would therefore be beneficial for the implementation of personalized therapies where the effect of the drugs would only be elicited at desired time points. 

### 3.5. Use of Biomedical Engineering Tools in Intervention Design

As described, one of the challenges in the design of interventions in chemotherapeutic treatment is the decision on the dosage of drugs to be used for them to exhibit their optimal effects. The development of novel technologies that serve as a platform for monitoring the real-time effects of drug treatment on human cells or tissues would therefore be of great value. Previously, Kleinhans et al. had developed a sensor-based methodology in monitoring the effect of the treatment of various dosages of chemotherapeutic drugs on the metabolic profile of tumor tissue explants [68]. In the study, the authors developed sensors that enable the detection and measurement of the level of oxygen uptake and pH, both of which would indicate the level of activity of the cellular respiration pathway. In addition, it helps the detection of the “Warburg effect” [69], which is featured by increased lactate production, which characteristically occurs in tumors, due to alterations in pH caused by lactate production. This pioneering work would potentially lead to the development of wearable sensors for patients, which their metabolic status can be measured, thus enabling the precise monitoring of the metabolic profile of the patients once chemotherapeutic drugs are administered. In addition, digital applications for use in mobile phones may be developed in connection with these wearable sensors [70], so that readings taken at different time points can be recorded, enabling the determination of the trends in the changes of the metabolic profile in response to the administered drugs. Such monitoring could potentially help to determine whether drug dosage needs to be modified based on the level of effect of the administered drugs on the metabolic rate of the patients.

One potential application of wearable sensors in personalized medicine would be the development of these sensors and digital applications in monitoring the metabolic profile of breast cancer patients with tamoxifen-resistant tumors. A previous study revealed that tamoxifen resistance in tumors can be mediated by the increased activity of glyoxalase, which is an enzyme that detoxifies methylglyoxal, a toxic by-product of glycolysis [71]. In other words, tamoxifen resistance could be associated with the lowered level of oxidative stress. Therefore, personalized therapies for these patients may involve the administration of chemotherapeutic drugs, combined with chemicals that increase reactive oxygen species (ROS) production to generate oxidative stress. The normality of cellular metabolism of the patients can then be monitored by the wearable sensors to ensure that the side effects of the therapy, which are caused by the addition of pro-oxidant chemicals, are kept minimal. This monitoring may further be coupled by the use of novel high-quality imaging technologies for the detection of breast tumors, thereby enabling the monitoring of breast cancer recurrence [72].

### 3.6. The Use of Genomic-Adjusted Radiation Dose (GARD) in Optimizing Radiation Dose in Radiotherapy

Recently, a retrospective, cohort-based study [73] reported the potential use of genomic-adjusted radiation dose (GARD) as a means in personalizing the radiation dose that is used in radiotherapy. The study involves 20 disease sites, including two breast cancer cohorts with survival data. They demonstrated that GARD shows an association with the clinical outcome of radiotherapy, indicating that GARD could potentially serve as a predictor of the outcome of patients receiving radiotherapy. Nevertheless, there are no clear cut experiments that demonstrate that this approach could indeed be translated clinically as a predictive biomarker, due to the current lack of studies that use radiation therapy versus control (no radiation) to examine the additional benefit of radiation treatment to those deemed responsive to radiation, as discussed by Spratt et al. [74]. Despite this, these findings would be useful to provide a framework for personalized medicine involving radiation therapy by taking account of the biological heterogeneity of cancer genomics into the design of better gene expression based and radiosensitivity index scores-guided clinical trials of therapeutic strategies.

## 4. Recommended Strategies of Personalized Medicine Development in Improving the QOL of Patients Undergoing Chemotherapy

As discussed, the use of chemotherapeutic drugs is often associated with the experience of adverse side effects that would drastically reduce the QOL and performance status of cancer patients [75]. In light of this, personalized medicine strategies should not be limited to the enhancement of the efficacy of the treatment process. They should also address the design of interventions that target the aforementioned side effects of cancer treatment. Here, we focus on how our current knowledge on genomics and metabolomics may help to achieve the development of personalized interventions that improve the QOL of breast cancer patients during and after chemotherapy treatment, and describe the findings that would help develop novel strategies for personalized therapies for breast cancer patients to improve their QOL.

### 4.1. Targeting Premature Menopause and CIM

Premature menopause is generally caused by the declining of the functions of the ovaries in women aged 40 or below, due to a deficiency in estrogen production [76]. Therefore, the search for genetic polymorphisms that are associated with biosynthesis and metabolism of estrogen would provide useful information on genetic biomarkers that can be targeted to address premature menopause or CIM in breast cancer patients. Efforts had previously been made to pinpoint the genes that can be targeted for therapeutic development to address premature menopause and CIM, primarily through association studies between genetic polymorphisms and sex hormone production. Nevertheless, earlier studies failed to identify any genetic polymorphisms that demonstrate this association [77,78]. Although Riancho et al. did demonstrate that certain sex hormone binding globulin (*SHBG*) gene polymorphisms are associated with serum SHBG levels, these gene polymorphisms were shown to not be associated with fragility bone fractures, which is one major symptom of premature menopause [78]. Nevertheless, a more recent study among a Brazilian population demonstrated that single nucleotide polymorphisms (SNPs) in the genes coding for estrogen receptors (*ESR1* and *ESR2*) were associated with premature ovarian failure, a feature of premature menopause [79]. This finding therefore suggests novel allele biomarkers that can be targeted for the treatment of breast cancer patients with premature menopause. It also provides a basis for further research into the mechanisms of how SNPs may lead to premature menopause and/or CIM. This would yield useful clues on the development of personalized therapeutic strategies, which may involve the incorporation of these therapeutic strategies into the existing chemotherapeutic treatment regimen, in the management of the symptom among patients.

However, even though premature menopause is generally considered as an undesirable side-effect, it is noteworthy that patients with amenorrhea, one clinical feature of premature menopause [76], was reported to exhibit better outcomes, including increased overall survival and disease-free survival rates, after chemotherapy [80]. Thus, future studies evaluating personalized treatment regimens targeting premature menopause and its associated symptoms should balance the pros and cons of premature menopause in terms of treatment efficacy by taking the above observation into consideration. 

### 4.2. Targeting CIPN

Recent studies have also identified several genetic biomarkers that are associated with CIPN, yielding useful clues as to how neurotoxicity induced by chemotherapeutic drugs can be reduced and managed among cancer patients undergoing chemotherapy. Kus et al. found that the 3435 TT genotype of *ABCB1*, a gene coding for a protein belonging to the ATP binding cassette subfamily, would lead to a significant increase in the risk of neurotoxicity among breast cancer patients undergoing chemotherapy with paclitaxel and docetaxel [81]. Likewise, the gene coding for a Charcot-Marie-Tooth protein, NDRG1, was suggested to be a genetic biomarker for CIPN, due to the negative correlation between the expression level of the gene and severity of CIPN [82]. Additionally, carriers of the CYP2C8*3 and/or FGD4 c.2044-236 G > A polymorphisms were also shown to be at greater risk of CIPN, leading to a requirement of reducing paclitaxel dose at an early stage of chemotherapy among these patients [83]. Overall, the aforementioned genes can be served as novel gene targets for addressing the neurotoxicity that is induced by chemotherapeutic drugs, which would be of value for the development of interventional strategies for CIPN management.

### 4.3. Targeting Cognitive Dysfunction

Chemotherapy treatment was known to be a causal factor for the development of cognitive impairment among cancer patients. Although the exact mechanism is yet unknown, inflammation had been suggested to be one of the potential mechanisms [84]. In line with this, a recent study showed that one polymorphism in the *IL1R1* gene, whose gene product plays a role in promoting inflammation, is associated with a higher degree of perceived cognitive function among breast cancer survivors [85]. This finding has therefore identified a possible biomarker to identify individuals undergoing chemotherapy who would be at a reduced risk of cognitive dysfunction. Personalized interventions targeting cognitive impairments may be implemented among those at higher risk of cognitive dysfunction only, thereby saving resources for intervention implementation.

### 4.4. Targeting Depressive Symptoms

Depression is one of the psychological symptoms that appears to form symptom clusters with other major symptoms that are experienced by breast cancer patients, including pain, fatigue, and sleep disturbance [86,87], and it was shown to be associated with poor emotional, functional, physical, and social well-being of breast cancer patients undergoing adjuvant cancer therapy [38]. Therefore, personalized therapies targeting depression could be an effective strategy in improving the QOL of breast cancer patients. In a recent study [88], the gene coding for brain-derived neurotrophic factor (BDNF) was suggested to be a gene biomarker for depression, thereby providing an alternative route to the early identification of breast cancer patients who can be targeted for therapies against depression, which would result in better outcomes. The authors identified a genetic polymorphism in the *BDNF* gene, which results in a substitution of Val66 with a methionine, is significantly associated with the seral level of C-reactive protein (CRP), and it predicts the severity of cognitive depression. These findings therefore indicate that the Val66Met polymorphism in the BDNF gene could be used as an early determinant on whether patients should be subjected to anti-depression therapies, enabling them to receive such treatment before depressive symptoms appear. Moreover, it has been reported that the symptoms that are associated with depression among cancer patients can be considerably different from those among healthy individuals [89], suggesting that the symptomatology of depression could vary between persons. In light of this, patients that are diagnosed with the Val66Met polymorphism of the *BDNF* gene should be further screened for the set of symptoms experienced by these patients, and treatment strategies should be tailored to the needs of each individual patient, based on the symptoms that are observed among these patients. This strategy of personalized therapy could be effective in ensuring that the detrimental effect of depression on the QOL of susceptible patients can be kept minimal. 

### 4.5. Targeting Pain

As discussed, pain is one of the most common symptoms among cancer patients, and this is primarily due to the side effect of the cancer treatment the patients receive. The identification of new biomarkers that are associated with pain as a result of cancer treatment would prove useful in the development of new personalized therapeutic strategies in pain management among patients. Recently, studies had pointed towards the possibility that the reduction of breast cancer-related lymphedema, the use of antioxidants, and that of nicotinamide riboside could be the potential routes to the development of personalized therapeutic strategies in targeting pain relief.

Lymphedema, a painful complication that is characterized by swelling of the arms and legs as a result of breast cancer treatment, is one of the most common symptoms that is experienced by breast cancer patients and survivors [90]. A recent study demonstrated that breast cancer survivors with lymphedema have a higher level of total polyunsaturated fatty acids (PUFA), increased activity of fatty acid desaturase, and an increased ratio between arachidonic acid and eicosapentaenoic acid [91]. These findings therefore suggest a potential relationship between the extent of fatty acid metabolism and lymphedema risk. Further, these findings could suggest that PUFA status of patients could serve as a biomarker for targeting pain that is experienced by breast cancer patients, and indicate the possibility that modulating PUFA intake could be an effective strategy in reducing lymphedema-associated pain among breast cancer patients. Limitation in polyunsaturated fatty acid intake in the diet of these patients could therefore serve as a personalized strategy in the treatment of patients that are suffering from lymphedema, despite the need for further studies with breast cancer patients of various ethnicities to confirm the findings by Ryu et al. [91].

Therapies using antioxidants were also suggested to be one potential effective strategy to target neuropathic pain caused by chemotherapy treatment. Indeed, oxidative-stress-associated mitochondrial dysfunction was found to be a possible mechanism mediating the development of neuropathic pain that is induced by chemotherapeutic drugs [92], and this potentially suggests that mitochondrial dysfunction could also be a biomarker that can be targeted to develop new therapies against pain. Consistent with this, Galley et al. showed that melatonin, a powerful antioxidant, can decrease the extent of mitochondrial damage and cause relief in neuropathic pain in rats that are administered with paclitaxel [92]. This study therefore provides us with an alternative strategy in the management of chemotherapy-induced neuropathic pain among cancer patients undergoing chemotherapy, which can be personalized for patients with CIPN.

Nevertheless, controversies do exist regarding the effectiveness of the use of antioxidants in conjunction with cancer therapies. Therefore, whether antioxidant supplementation should be provided to the cancer patients undergoing cancer therapies is still currently a subject of debate [93]. While antioxidants serve to reduce the level of free radicals, chemotherapy and radiotherapy naturally rely on the cytotoxic action of free radicals on tumors in order to exhibit their treatment effect. Therefore, it was generally perceived that antioxidant supplementation in combination with chemotherapy and radiotherapy would compromise the effectiveness of these therapies, thereby affecting patient outcomes [94]. However, increased survival and enhanced QOL were observed among patients who received a combination of radiotherapy and supplementation of melatonin, a hormone that exhibits antioxidant properties [95]. Similar observations were made in a randomized clinical trial, reporting a significantly better overall survival among non-small cell lung cancer patients who received radiotherapy with the supplementation of alpha-tocopherol, a known antioxidant [96]. These controversies could perhaps be explained by the counteractive nature of the cyto-protective effect of antioxidants and the cytotoxic effect of cancer therapies. Moreover, it has also been suggested that the dosage of antioxidants used, the duration of supplementation, and the route of administration of the antioxidants could lead to variations in the effect of the antioxidants in patient outcomes [93]. Indeed, it was suggested that the antioxidant alpha-tocopherol would turn into a pro-oxidant if supplemented in high concentrations [97]. In light of the varied effect of antioxidant supplementation on patient outcomes, further large-scale clinical trials need to be conducted to investigate the effect of the differences in dosage and route of administration of antioxidants, and that in the duration of antioxidant supplementation, on the outcomes of patients undergoing cancer therapies.

Nicotinamide riboside (NR) serves as a precursor molecule for the generation of nicotinamide adenine dinucleotide (NAD), which is a coenzyme that was previously known to protect against neuronal degeneration that is attributed to neurotoxicity [98]. Recently, Hamity et al. demonstrated that treatment of rats with NR would greatly ameliorate the level of tactile hypersensitivity after paclitaxel administration, and reduce their place escape/avoidance behaviors [99], demonstrating the potential of NR in being used as alternative personalized treatment for chemotherapy-induced neuropathic pain among cancer patients. The mechanism of how NR can contribute to reduction in pain is yet unclear. However, a recent study showed that the increase in NAD levels, an effect that is identical to NR treatment, by a naturally-occurring anthocyanin would lead to the inhibition of inflammasome activation and pro-inflammatory gene expression through the ablation of the nuclear translocation of nuclear factor-κB (NF-κB) and the inhibition of nucleotide oligomerization domain protein 1/2 signalling [100]. This therefore suggests that the potential effect of NR treatment in pain relief could be mediated through the reduction of inflammation, although further studies are required to confirm this. Nevertheless, therapies using NR, alongside those using melatonin, could prove a useful strategy in planning personalized treatment targeted for patients with CIPN.

### 4.6. Additional Targets for Personalized Therapies

Although obesity is not considered to be a side effect of chemotherapy for breast cancer treatment, the finding that increases in body weight after breast cancer diagnosis is associated with higher risk of cancer recurrence [101] suggests that implementation of interventions aiming to prevent obesity among breast cancer patients is still warranted. Recently, an association study was carried out demonstrating the association of SNPs of certain genes with changes in body mass index (BMI) among breast cancer survivors. These genes all code for proteins that are involved in fat metabolism, which include *ADIPOR1* (coding for adiponectin receptor 1, which takes part in signaling pathways involved in regulation of fatty acid oxidation), *FTO* (coding for fat mass and obesity-associated protein, which is implicated in obesity) and *FNDC5* (coding for a hormone for white fat conversion to brown fat) [102]. The identification of these SNPs that are associated with weight gain among breast cancer survivors has revealed novel biomarkers that can be used in identifying individual breast cancer patients or survivors who would are in need of certain interventions that prevent them from being obese. The inclusion of interventions, such as those for lifestyle modifications to lower obesity risk in personalized cancer treatment for breast cancer survivors, would therefore reduce their risk of breast cancer recurrence. These interventions could include exercise intervention, which was previously shown in a systematic review to be feasible and acceptable to cancer patients undergoing neoadjuvant cancer treatment, and to be effective in improving their physical fitness [103].

## 5. Conclusions

As a result of the unpleasant side effects of the currently practiced cancer treatment regimens, some breast cancer patients experience undesirable cancer-related symptoms during the process of cancer treatment. This potentially results in the reduced drug dosage used during treatment or even treatment cessation, rendering the treatment process ineffective. Personalized therapies, treatment that is provided according to the needs of individual patient, could potentially address this issue. Two strategies key to the development of personalized therapies are: (1) optimization of drug choice or dosage used in treatment; and (2) identification of genetic changes that are associated with cancer symptom occurrence and severity.

Recent studies have revealed a number of genomic changes (including SNPs and gene overexpression) and metabolomic changes that would lead to differences in the efficacy of the treatment and effect on symptom severity and QOL in different individuals. This thereby enables us to identify certain genetic biomarkers that can be used as tools for the determination of the optimal drug choice or dosage to be used in cancer treatment. This would enable the treatment process to achieve its required efficacy, yet it would not cause too much discomfort for patients undergoing such treatment. Furthermore, with the development of novel techniques and technologies, such as the use of microRNA for timely chemotherapeutic drug release and wearable sensors to detect any abnormal changes in metabolic rate of patients as a result of drug administration, the side effects that are caused by the treatment process itself could potentially be significantly ameliorated.

Gene profiling studies would also help to identify the genetic biomarkers that can predict the risk of individuals to develop common symptoms that are associated with cancer treatment. Studies on the metabolic changes that are associated with the occurrence and severity of certain cancer-associated symptoms have also helped identify a number of molecular candidates that can be used as determinants on whether patients are at higher risk of increased severity of a particular symptom, and whether these candidates can be targeted for symptom management in patients. All of these would inform the development of novel strategies in planning for personalized therapies in symptom amelioration, thereby ensuring a better QOL for patients undergoing cancer treatment.

Despite recent advances in the identification of novel biomarkers that affect treatment efficacy and symptom severity, the molecular mechanisms of how they exert their effects is still not fully understood. Furthermore, confirmation on the findings of the current studies on biomarker identification, through additional studies on the association of these biomarkers and treatment efficacy and symptom severity among breast cancer patients of various ethnicities, is generally lacking. Further studies on these issues are therefore warranted in order to enable the exploration and development of further strategies that can be utilized in optimizing cancer therapies for breast cancer patients, thereby augmenting the effectiveness of cancer treatment and improving the QOL of patients during the treatment process.

## Figures and Tables

**Table 1:** The classification of breast cancer sub-types based on the expression of biomarkers in tumors. ER: estrogen receptor; HER2: human epidermal receptor 2; Ki-67: a protein marker for proliferation; PR: progesterone receptor.

Breast Cancer Type	ER	HER2	Ki-67	PR
Luminal A	Positive	Negative	Low level	High level
Luminal B	Positive	Negative	High level	Low level
HER2-positive	Negative	Over-expressed	Unclear	Negative
Triple negative	Negative	Negative	Unclear	Negative
